# Identifying Voice Individuality Unaffected by Age-Related Voice Changes during Adolescence

**DOI:** 10.3390/s22041542

**Published:** 2022-02-17

**Authors:** Natsumi Suzuki, Momoko Ishimaru, Itsuki Toyoshima, Yoshifumi Okada

**Affiliations:** 1 Division of Information and Electronic Engineering, Muroran Institute of Technology, 27-1, Mizumoto-cho, Muroran 050-8585, Hokkaido, Japan; 20043028@mmm.muroran-it.ac.jp (N.S.); 21043010@mmm.muroran-it.ac.jp (M.I.); 21043048@mmm.muroran-it.ac.jp (I.T.); 2 College of Information and Systems, Muroran Institute of Technology, 27-1, Mizumoto-cho, Muroran 050-8585, Hokkaido, Japan

**Keywords:** voice individuality, speaker verification, Fisher’s F-ratio, adolescence, deep learning

## Abstract

Identifying voice individuality is a key issue in the biometrics field. Previous studies have demonstrated that voice individuality is caused by differences in the shape and size of the vocal organs; however, these studies did not discuss voice individuality over a long term that includes periods of voice change. Therefore, we focus on adolescence (early teens to early twenties), which includes voice changes due to growth of vocal organs, and we reveal invariant voice individuality over a long period. In this study, the immature and mature periods during vocal organ development were defined as unstable and stable periods, respectively. We performed speaker verification tests across these two periods and evaluated voice features that are common to these periods using Fisher’s F-ratio. The results of the speaker verification test demonstrated a verification accuracy of 60% or more in most cases, and the results of the evaluation using Fisher’s F-ratio demonstrated that robust voice individuality existed in the frequency regions of 1–2 kHz and 4–6 kHz regardless of the period. These results suggest that voice individuality is unaffected by age-related changes over the long term, including adolescence.

## 1. Introduction

Biometrics technologies are used to identify and authenticate individuals based on their physical characteristics. To date, many biometrics techniques that use physical characteristics, e.g., face, voice, fingerprint, iris, gait, and palmprint, have been proposed [1,2,3,4,5,6,7,8]. Among such techniques, speaker verification is easy to implement and can be used to authenticate people remotely using telephone and video calls. Speaker verification discriminates differences between individuals, i.e., voice individuality, using voice features, e.g., the frequency spectrum and sound spectrogram of the voice [9,10,11,12]. Thus, identifying individuality in voice features is very important in terms of achieving accurate speaker verification systems.

To date, many studies of voice individuality have been presented [13,14,15,16,17,18,19]. Most focused on the frequency characteristics produced by the resonance of the speaker’s vocal tract [15,16,17,18,19]. Some studies have revealed that voice individuality and the shape and size of the vocal organs are closely related [17,18,19]. However, to the best of our knowledge, no study has investigated voice individuality relative to age-related changes due to vocal organ growth. As the shape and size of the vocal organs change, voice characteristics also change [20]. Thus, a change in voice characteristics can reduce verification accuracy. Therefore, it is important to identify voice individuality in a manner that is robust against age-related changes due to vocal organ growth.

The goal of this study was to reveal invariant voice individuality during adolescence, which is the period in which vocal organs grow significantly. According to the World Health Organization [21], adolescence is defined as the phase of life between childhood and adulthood (ages 10 to 19). During puberty, which occurs during the first half of adolescence, the voice changes because the vocal organs grow significantly with secondary sexual characteristics. Thus, we expect that identifying voice individuality in adolescence will help reveal voice individuality information that is robust against long-term age-related changes.

In this study, experiments were performed using Japanese audio data of voice actors (early teens to early twenties) who have appeared continuously in a series of movies over a period of 10 years. The audio data were divided into an immature (i.e., unstable) period and mature (i.e., stable) period based on the developmental stage of the vocal organs. To clarify voice individuality common to these two periods, we conducted two experiments using phrase samples and vowel samples from the audio data. First, we performed speaker verification tests across the unstable and stable periods to determine whether target speakers can be identified correctly before and after vocal organ growth. Second, we evaluated the relationship between frequency bands and voice individuality using Fisher’s F-ratio to identify voice features that are robust and unaffected by age-related changes caused by growth of the vocal organs.

The remainder of this paper is organized as follows. Section 2 introduces related work. Section 3 explains the experimental method, and the experimental results are presented in Section 4. A discussion of the results is given in Section 5. Finally, conclusions and suggestions for future work are presented in Section 6.

## 2. Related Work

The differences in voice qualities between individuals are caused by differences in the characteristics of the vocal organs [22]. For example, the shape and size of the vocal tract and vocal cords play a very important role in voice individuality. Lu and Dang investigated the relationship between vocal organs and frequency components [17], and found that speaker identification information derived from vocal organs was concentrated in three frequency regions, i.e., the 100–300 Hz region (related to the length and stiffness of the vocal cords), the 4.0–5.5 kHz region (related to the shape of the piriform fossa), and the 6.5–7.8 kHz region (related to consonants). In addition, Kitamura et al. investigated the relationship between Japanese vowels and vocal individuality [18,19]. They found that vowel individuality was observed in the frequency region of approximately 2.5 kHz or higher and that this result was related to the shape of the hypopharynx, which comprises the laryngeal tube and piriform fossa. As mentioned previously, the shape and size of the vocal organs and voice individuality are closely related; however, these previous studies did not consider the relationship between vocal organ growth and voice individuality. Thus, in this study, we investigated voice individuality that is independent of age-related changes caused by the growth of vocal organs.

Voice individuality information is commonly used in speaker verification systems, and age-related voice changes must be considered when developing such systems. To date, several studies have investigated speaker verification techniques that are robust against age-related voice changes [23,24,25,26]. However, these studies did not consider the developmental periods in the growth process of vocal organs. In contrast, in this study, we focused on adolescence, which includes periods of voice change due to significant growth in the vocal organs. Specifically, we investigated the possibility of speaker verification between two different periods in adolescence, i.e., the unstable period, in which the vocal organs are immature, and the stable period, in which the vocal organs are mature.

## 3. Methodology

### 3.1. Definition of the Unstable Period and the Stable Period

The growth in vocal cord length and vocal tract length stops by the ages of 20 and 21, respectively, regardless of gender [27,28]. Thus, in this study, we defined the unstable period as less than 20 years old, the mixed period as 20 years old, and the stable period as 21 years old or older. In our experiments, we considered voice data from the unstable and stable periods to verify whether there exists voice individuality that is independent of age-related changes during these periods.

### 3.2. Data Preparation

#### 3.2.1. Collection of Audio Data

We considered Japanese audio data in our experiments. At the start of this study, no Japanese audio data had been recorded continuously over a long period (including adolescence to the early twenties). Thus, we collected Japanese-dubbed voices of eight British movies that were serialized over a 10-year period. We first selected six Japanese voice actors (three males and three females) who performed in the eight movies from their unstable periods to their stable periods. Then, we collected monaural audio data of each voice actor, in which utterances with sound effects or BGM were eliminated. Here the sampling rate was set to 16 kHz, and the quantization bit rate was set to 16 bits. We then divided the audio data of each voice actor into the unstable and stable periods.

Table 1 shows the details of the six voice actors. One male voice actor and one female voice actor who played in all movies had the highest number of utterances, and these actors were selected as the verification targets. In this paper, the male and female voice actors are referred to as targets M and F, respectively. The remaining male and female voice actors are referred to as non-target M1, non-target M2, non-target F1, and non-target F2.

#### 3.2.2. Extraction of Vowel Data

**Figure 1:**
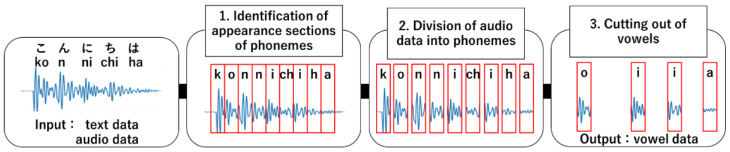
Extraction of vowel data.

The Japanese language has five vowels: /a/, /i/, /u/, /e/, and /o/. Here, we explain the method used to extract these vowels (hereafter referred to as vowel data) from the collected audio data. Here, Julius [29], which is a high-performance general-purpose engine for large vocabulary continuous voice recognition, was employed to extract the vowel data. An important function of Julius is the alignment output function, which identifies the appearance section of a phoneme by comparing the input audio data and their corresponding text data. The appearance sections of phonemes were identified using a Japanese acoustic model based on a hidden Markov model. Figure 1 shows the procedure used to extract the vowel data. First, the appearance sections of phonemes in the audio data were identified using Julius, and the audio data were divided into phonemes based on the identified appearance section. Then, only the appearance sections of vowels, i.e., vowel data candidates, were extracted from the phoneme data. As a result, vowel data candidates were collected automatically by performing these operations on all audio data. In addition, we manually selected clear vowels as the final vowel data.

#### 3.2.3. Preparation of Voice Samples

We prepared voice samples for our experiments using the collected audio data (Section 3.2.1) and the extracted vowel data (Section 3.2.2). The voice samples were extracted by shifting a window of a fixed frame width from the start point of the audio data and vowel data. Note that silent samples were eliminated. Here, the frame width and overlap width were set to 50 and 40 ms, respectively. In this paper, the voice samples obtained from the audio and vowel data are referred to as phrase samples and vowel samples, respectively. Table 2 shows the number of phrase and vowel samples in the unstable and stable periods.

### 3.3. Experimental Setup

#### 3.3.1. Extraction of Voice Feature

In most speaker verification systems, the mel-frequency cepstral coefficient (MFCC) is used as the baseline voice feature [30]. However, recent studies have suggested that the linear-frequency cepstral coefficient (LFCC) is superior to the MFCC as a voice feature for speaker verification tasks [31,32]. The MFCC focuses on only the low-frequency region; however, speaker characteristics are present in both the low-frequency and high-frequency regions [17]. LFCC transforms frequencies using a uniform linear frequency filter bank; thus, it can extract frequency characteristics uniformly without losing the characteristics of the speakers in high-frequency regions. Therefore, LFCC was used as the voice feature in our experiments.

**Figure 2:**
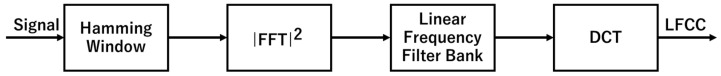
Extraction of LFCC.

Figure 2 illustrates the LFCC extraction procedure. For each generated voice sample (Section 3.2.3), the amplitude was first normalized to the range −1 to 1, and a Hamming window was applied. Then, Fast Fourier Transform (FFT) was applied, and the absolute values of the amplitudes after the FFT were squared to transform them into a power spectrum. Subsequently, the power spectrum was filtered using 60 linear frequency filter banks. Finally, a 60-dimensional LFCC was extracted by applying a discrete cosine transform (DCT).

#### 3.3.2. Construction of Verification Model

**Figure 3:**
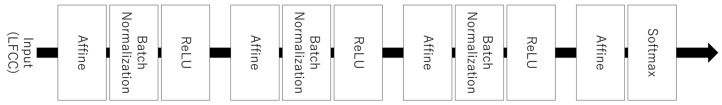
Architecture of verification model.

A deep neural network (DNN) is a multi-layer feedforward NN with at least three hidden layers [2]. In this study, we used a DNN to construct verification models for targets M and F. Figure 3 shows the architecture of the verification model. The input to the model is a 60-dimensional LFCC. Affine is a fully-connected layer in which each node connects to all nodes of the subsequent layer. Batch Normalization [33] was introduced to reduce overfitting and achieve faster learning. ReLU is a function that was implemented to avoid the vanishing gradient problem and achieve faster learning. Softmax is a function that was introduced to output the probability distribution over all classes. In this experiment, the number of training iterations was set to 300, and the mini-batch size was set to 32. The verification model was trained to identify two classes, i.e., a target class (a set of voice samples of either target M or F) and a non-target class (a set of voice samples of the four non-targets). During training of the verification model, the parameters were updated via backpropagation. Cross entropy was used as the loss function, and Adam [34] was used as the optimization method. In the verification test, the probabilities, which were calculated by the Softmax function, of the target class and non-target class were output in the range of 0 to 1 for the input test data.

#### 3.3.3. Evaluation Index in Verification Test

**Figure 4:**
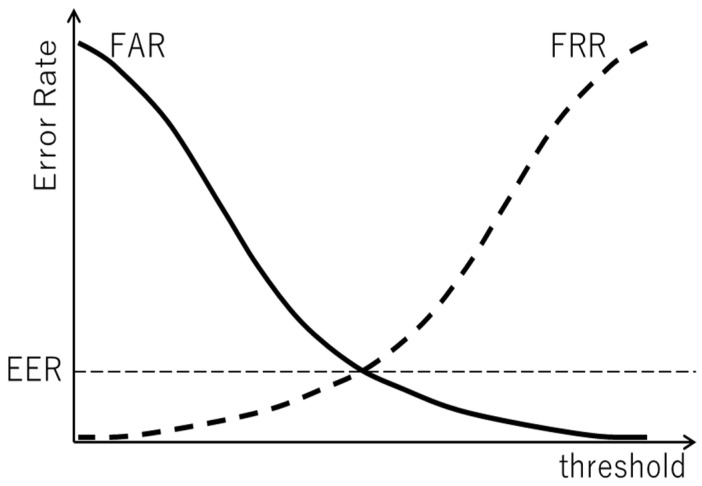
Calculation of EER using FAR and FRR.

We employed an index based on the equal error rate (EER) [35] to evaluate the verification accuracy for the target class. For the probability of the target class output from the DNN, the thresholds were set to the values in the range 0 to 1 in increments of 0.01. The following operations were executed for the respective thresholds to calculate the EER. First, using the DNN, the input data were classified as the target class if the corresponding probability exceeded a given threshold; otherwise, the input data were classified as the non-target class. Then, the false acceptance rate (FAR) and false rejection rate (FRR) were computed using the classification results. FAR and FRR are defined as follows:

(1) $$\mathrm{FAR}=\frac{\mathrm{FP}}{\mathrm{FP}+\mathrm{TN}},$$

(2) $$\mathrm{FRR}=\frac{\mathrm{FN}}{\mathrm{FN}+\mathrm{TP}},$$

where FP is the number of times the non-target was classified incorrectly as the target, TN is the number of times the non-target was classified correctly, FN is the number of times the target was classified incorrectly as the non-target, and TP is the number of times the target was classified correctly. After executing these operations for all thresholds, the EER was calculated using the FAR and FRR values. As illustrated in Figure 4, there is a trade-off between FAR and FRR. The EER was calculated as follows using the FAR and FRR values when those values matched (or were as closed as possible) at a given threshold value:

(3) $$\mathrm{EER}=\frac{\mathrm{FAR}+\mathrm{FRR}}{2}.$$

The final verification accuracy was evaluated using the EER as follows:

(4) Verification accuracy=1−EER.

### 3.4. Experimental Method

#### 3.4.1. Speaker Verification Test

**Figure 5:**
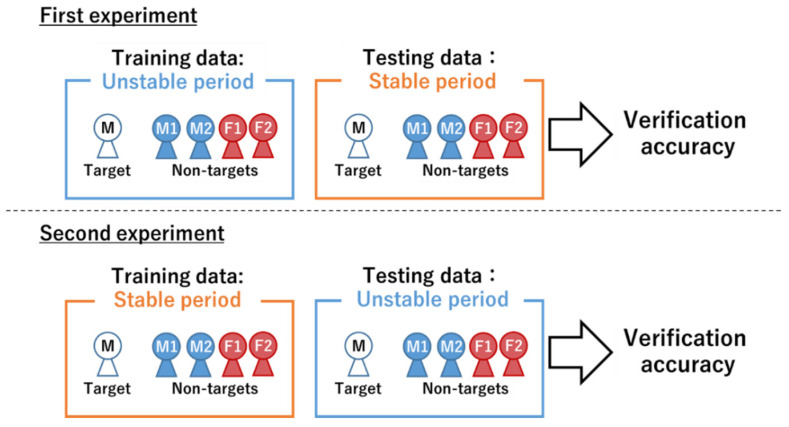
Speaker verification method.

To investigate the feasibility of speaker verification using voices that have changed over time, verification tests for targets M and F were conducted using voice samples of the six voice actors from both the unstable and stable periods. As shown in Figure 5, we conducted two experiments using the constructed DNN-based verification model (Section 3.3.2). The first experiment was conducted to confirm whether the voice samples from the stable period can be recognized correctly when using voice samples from the unstable period as training data. The second experiment was conducted to confirm whether voice samples from the unstable period can be recognized correctly when using voice samples from the stable period as training data. In these experiments, by changing the initial values of the weights and mini-batch sampling in the DNN, we generated five verification models for each of the targets M and F. Here, the average of the verification accuracy obtained using each verification model was used as the final verification accuracy.

#### 3.4.2. Evaluation of Voice Individuality

Lu and Dang investigated the relationship between each frequency band and speaker individuality using Fisher’s F-ratio (hereafter F-ratio) [17]. Following their work, we investigated the frequency bands involved in voice individuality relative to targets M and F. Here, the F-ratio was calculated as follows. First, a linear frequency filter bank was applied to the power spectrums calculated from the voice samples, and those power spectrums were divided into 60 frequency bands. Next, for each frequency band *k*, the average power spectrum $\mu_{i,s,k}$ of voice actor *i* in period *s* and the average power spectrum $\mu_{s,k}$ of all voice actors in period *s* were calculated as follows:

(5) $$\mu_{i,s,k}=\frac{1}{N_{i,s}}\sum_{j=1}^{N_{i,s}}x_{i,s,j,k\;},$$

(6) $$\mu_{s,k}=\frac{1}{I}\sum_{i=1}^{I}\mu_{i,s,k},$$

where $N_{i,s}$ is the total number of voice samples of voice actor *i* in period *s*, $x_{i,s,j,k}$ is the value of the power spectrum in frequency band *k* of voice sample *j* of voice actor i in period *s*, and *I* is the total number of the voice actors. The F-ratio of frequency band *k* for voice actor *i* in period *s* was calculated as follows:

(7) $${F\text{-}\mathrm{ratio}}_{i,s,k}=\frac{\frac{1}{I}\sum_{i=1}^{I}{\left(\mu_{i,s,k}-\mu_{s,k}\right)}^{2}}{\frac{1}{N_{i,s}}\sum_{j=1}^{N_{i.s}}{\left(x_{i,s,j,k}-\mu_{i,s,k}\right)}^{2}},$$

where the denominator and numerator indicate the intra-speaker and inter-speaker variances, respectively. Lastly, F-ratios were normalized using min-max normalization. A frequency band with a large F-ratio value can be considered a feature that effectively contributes to the discrimination of targets M and F, i.e., it represents voice individuality.

## 4. Results

### 4.1. Results of Verification Tests

The verification tests were conducted to determine whether it is possible to identify the voice of targets M or F between the unstable period and stable period. In the following, we present the verification accuracies obtained using the phrase and vowel samples.

#### 4.1.1. Verification Results Obtained with Phrase Samples

**Figure 6:**
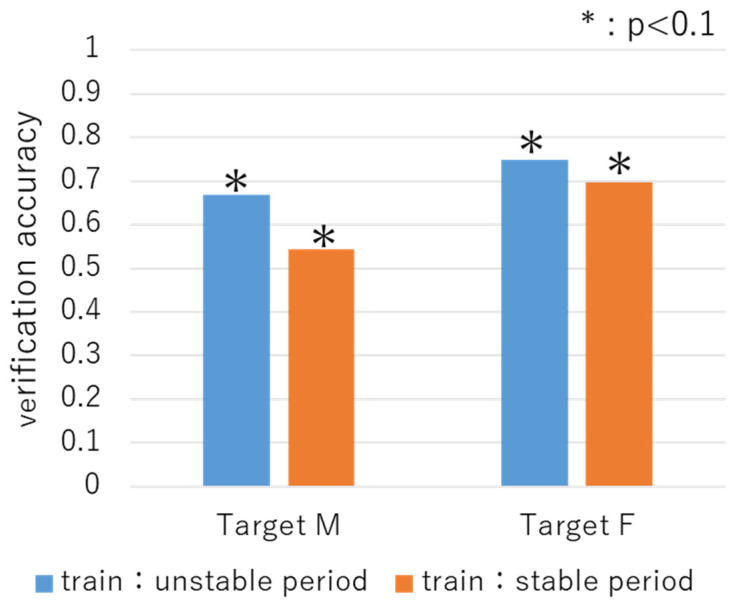
Verification accuracy obtained using phrase samples.

Figure 6 shows the verification accuracies for targets M and F obtained using the phrase samples. Here, the blue bar is the result in which the phrase samples in the unstable period were trained and the phrase samples in the stable period were tested. The orange bar is the result in which the phrase samples in the stable period were trained and the phrase samples in the unstable period were tested. As can be seen, the verification accuracy ranged from 54% to 75%, which is high compared to the expected values (*p* < 0.1). These results indicate that it is possible to identify the targets across the unstable and stable periods using the phrase samples. In addition, for both targets, we found that the verification accuracy was higher when the phrase samples of the unstable period were used for training.

#### 4.1.2. Verification Results Obtained with Vowel Samples

**Figure 7:**
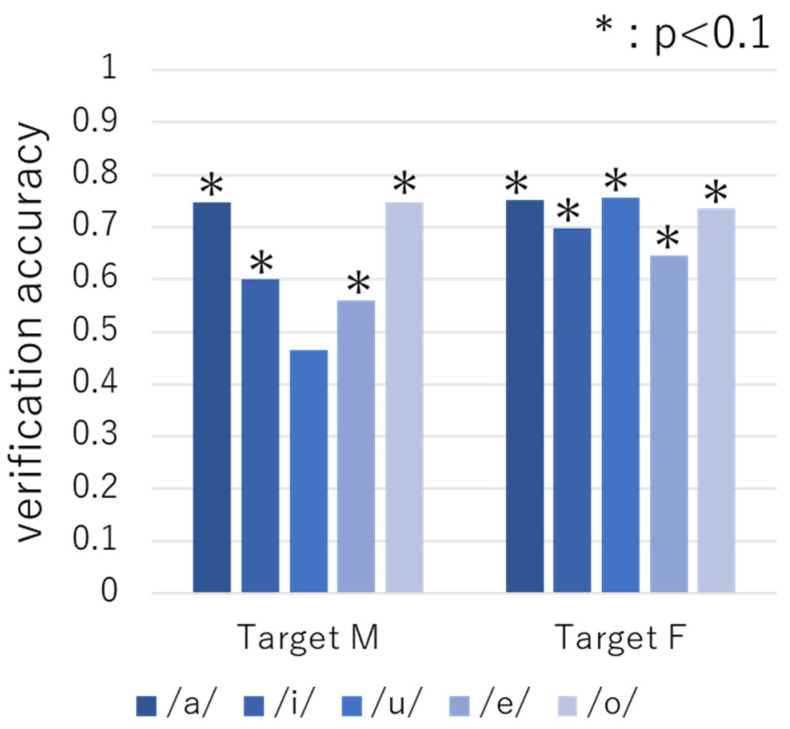
Verification accuracy obtained using vowel samples in the unstable period as training data.

**Figure 8:**
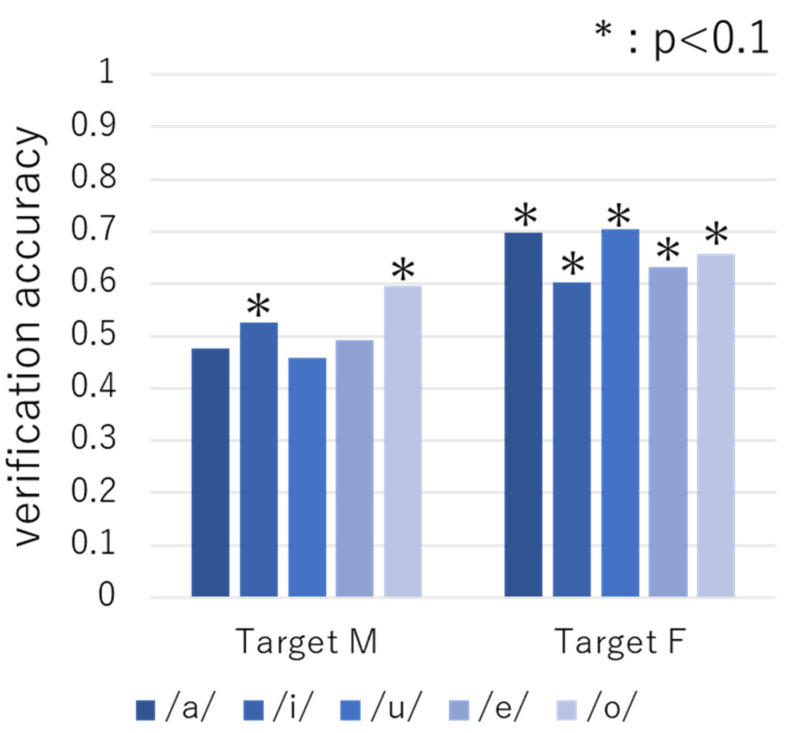
Verification accuracy obtained using vowel samples in the stable period as training data.

Figure 7 and Figure 8 show the results obtained when the vowel samples in the unstable and stable periods were used as training data, respectively. As can be seen, the verification accuracy ranged from 46% to 75%, and 13 out of the 20 cases exhibited 60% or more accuracy, which is high compared to the expected values (*p* < 0.1). These results indicate that it is possible to identify the targets across the unstable and stable periods using the vowel samples. In addition, similar to the results obtained using the phrase samples, we found that the verification accuracy was higher for both targets when training the vowel samples of the unstable period.

### 4.2. Frequency Bands Involved in Voice Individuality

As discussed in Section 3.4.2, the F-ratio allows us to identify the frequency bands that effectively contribute to speaker verification. In other words, a frequency band with high F-ratio can contribute to voice individuality. Here, the F-ratio was used to evaluate individual characteristics shared between the unstable and stable periods.

#### 4.2.1. Evaluation Results of Voice Individuality with Phrase Samples

**Figure 9:**
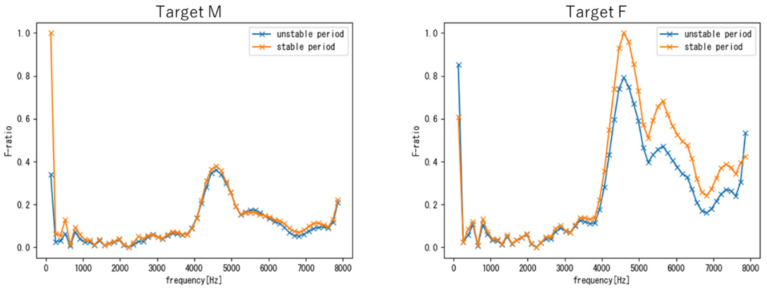
F-ratios obtained using phrase samples of targets M (**left**) and F (**right**).

Figure 9 shows the F-ratio obtained when using the phrase samples, where the left and right figures show the results for targets M and F, respectively. As can be seen, for both targets M and F, the frequency region around 4–6 kHz exhibited high F-ratios for both periods. In other words, this frequency region is likely to include individual voice characteristics that are common to the unstable and stable periods.

From the comparison between targets M and F, we can see that in the low-frequency band, the difference between the two periods in target M was larger than that of target F. This is because the fundamental frequency of males fluctuates greatly during the period of voice change.

#### 4.2.2. Evaluation Results of Voice Individuality Obtain with Vowel Samples

**Figure 10:**
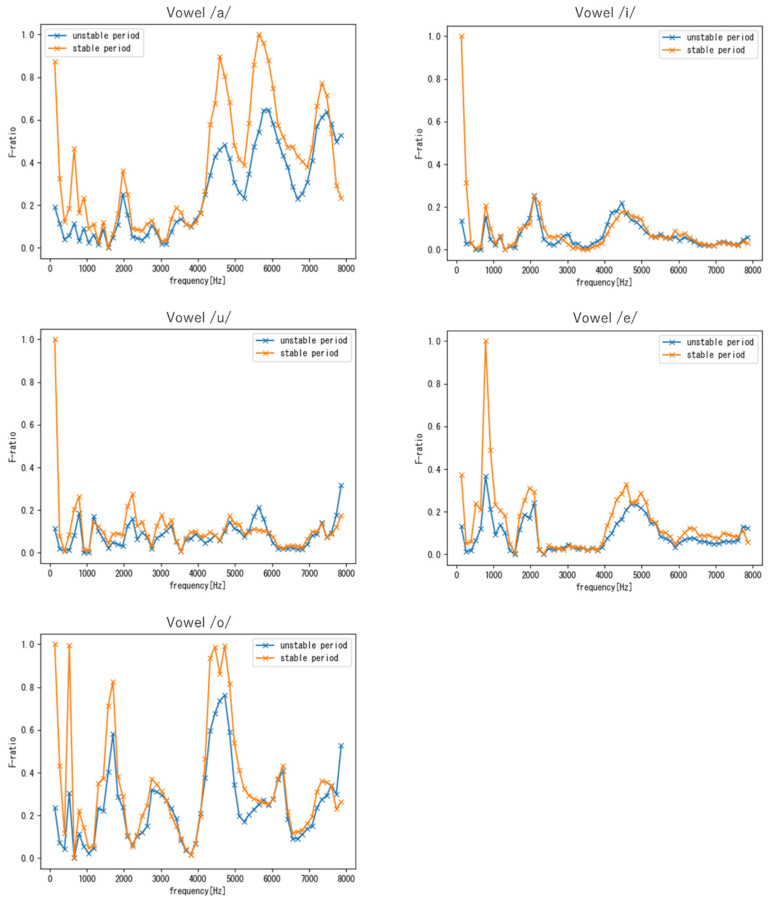
F-ratios of the five vowel samples (Target M).

**Figure 11:**
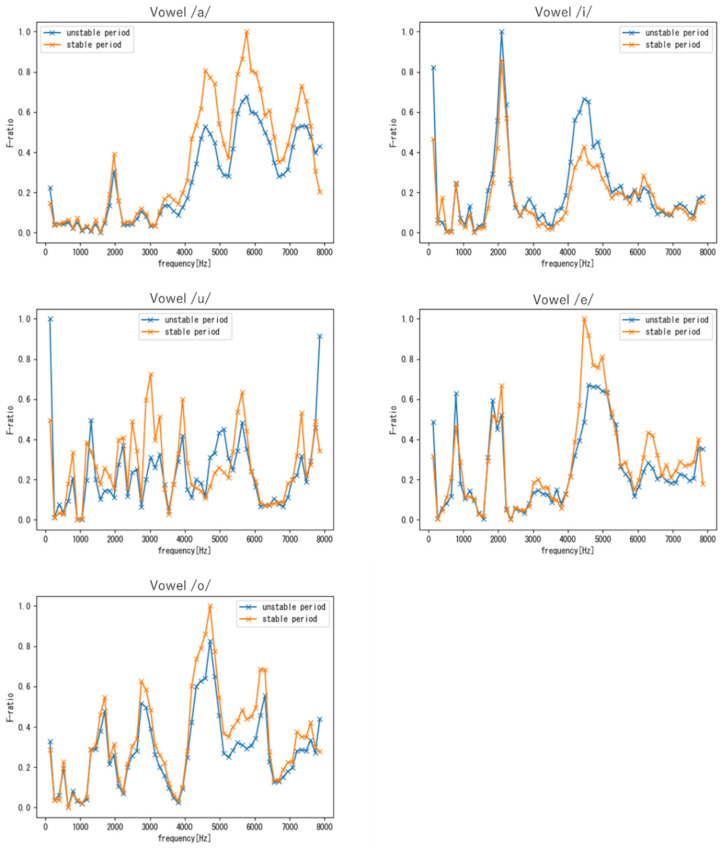
F-ratios of the five vowel samples (Target F).

Figure 10 and Figure 11 show the F-ratio results for targets M and F obtained using vowel samples, respectively. For the vowel /u/, no clear features were observed over the entire frequency range because, as shown in Table 2, the number of samples for vowel /u/ was extremely small compared to that of other vowels. Therefore, the results for the other vowels are discussed in the following. As shown in Figure 10 and Figure 11, similar to the results obtained with the phrase samples, the frequency region around 4–6 kHz exhibited high F-ratios in both periods. In addition, a frequency region with a high F-ratio was found to commonly exist around 1–2 kHz for all vowels. Note that this finding was unique to vowels, i.e., it was not observed with phrases. Furthermore, in addition to the 1–2 kHz and 4–6 kHz frequency regions, each vowel had unique frequency regions that demonstrated a common individuality in both the stable and unstable periods.

From the comparison between targets M and F, we can see that in the low-frequency band, the difference between the two periods in target M tended to be larger than that of target F for all vowels. As mentioned in Section 4.2.1, this is because the fundamental frequency of males fluctuates greatly during the period of voice change.

### 4.3. Evaluation of the Robustness of the Frequency Regions Related to Voice Individuality

In Section 4.1, it was shown that speaker verification was possible regardless of age-related voice changes using the DNN model. In Section 4.2, the frequency regions related to voice individuality were identified for each period. Here, we evaluated whether the two frequency regions identified in Section 4.2, 1–2 kHz and 4–6 kHz, show robustness with respect to age variations. This evaluation was conducted using the vowel samples of the targets and non-targets in the unstable period and stable period. We first transformed the vowel samples into the spectrograms. Next, for each spectrogram, we calculated medians of amplitude values in each frequency band. Subsequently, we generated pairs of two medians taken randomly from different two frequency regions. Finally, for each person (the targets and non-targets), we calculated the centroid of the pairs of medians. In this experiment, the centroids between the two periods for each person were compared to evaluate robustness of the 1–2 kHz and 4–6 kHz frequency regions in age variations.

**Figure 12:**
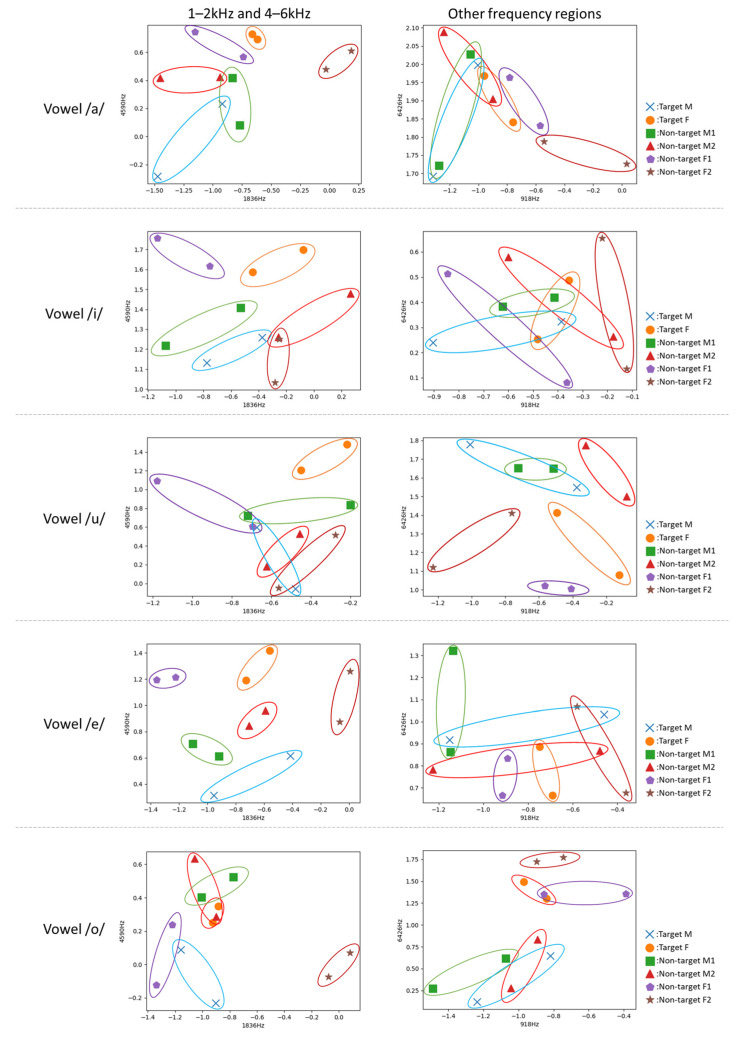
Scatter plots of the centroids of the unstable period and stable period.

**Figure 13:**
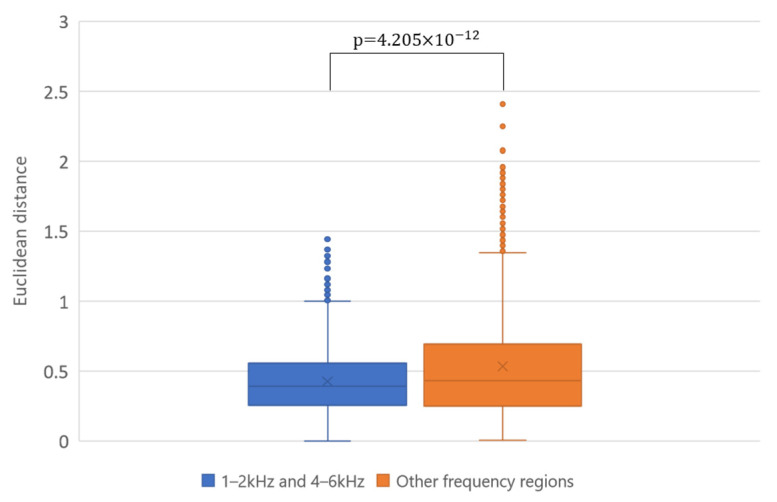
Boxplots for the Euclidian distances between the centroids of each person.

Figure 12 shows the scatter plots of the centroids. The markers of the same color in each figure indicate the centroids of the unstable period and stable period for each person. The figures on the left are scatter plots of the centroids calculated using medians taken from each of 1–2 kHz and 4–6 kHz. The figures on the right are scatter plots of the centroids calculated using medians taken from the frequency regions other than the 1–2 kHz and 4–6 kHz frequency regions. As can be seen, the results of the 1–2 kHz and 4–6 kHz frequency regions exhibited closer distances between the markers of the same person across the two periods compared to those of the other frequency regions. In addition, the markers of the two periods for each person formed a more distinct cluster in the results of 1–2 kHz and 4–6 kHz, especially in the vowels /a/, /i/, and /e/. In contrast, in the results of the other frequency regions, such a clear tendency was not observed. Figure 13 shows the boxplots for the distances (Euclidian distances) between the centroids for the two periods of each person. Each boxplot in this figure was created using 6000 centroids (i.e., 3000 distances) calculated from pairs of randomly obtained medians in the 1–2 kHz and 4–6 kHz frequency regions, or in other frequency regions. From this figure, we can see that the distance between the same person in the 1–2 kHz and 4–6 kHz frequency regions was significantly smaller than that in the other frequency regions. This means that the 1–2 kHz and 4–6 kHz frequency regions include voice individuality that is robust to age-related changes.

## 5. Discussion

As demonstrated by the results discussed in Section 4.1, verification accuracy was lower when the samples from the stable period were used as training data for both phrase and vowel samples. This is due to the influence of the number of samples. As discussed in Section 3.2.3, the number of samples in the stable period was less than half that in the unstable period. Therefore, we conjecture that sufficient training was not realized in the stable period.

In addition, the difference in verification accuracy between the unstable and stable periods was larger for target M than target F. Compared to females, males generally exhibit greater changes in voice pitch (i.e., in the fundamental frequency) from the unstable period to the stable period because vocal organ growth in males is greater during the unstable period than that of females. Accordingly, the intra-speaker variance in the fundamental frequency in males during the unstable period is also greater. In fact, as demonstrated in Section 4.2, the F-ratio of the low-frequency band that includes the fundamental frequency of target M was high in the stable period but low in the unstable period. In this case, a model trained using samples from the stable period is more likely to focus on the low-frequency band that possesses low discrimination power in the unstable period. To improve the verification accuracy of the model for the stable period, it will be necessary to remove the influence of the fundamental frequency.

The results presented in Section 4.1 reveal that it was possible to discriminate targets M or F across the unstable and stable periods. Thus, we consider that voice contains individuality that is unaffected by age-related changes in the period including adolescence. From the results presented in Section 4.2, we found that the F-ratio was high around the 4–6 kHz frequency region compared to other regions, which was a common characteristic in both the unstable and stable periods. Thus, this frequency region is likely to be involved in voice individuality. Lu and Dang [17] confirmed the existence of voice individuality in the 4.0–5.5 kHz frequency region, and they suggested that this region is related to the shape and size of the piriform fossa, which is a part of the vocal organs. In addition, Kitamura et al. [18,19] reported that voice individuality was present in a wide frequency region of approximately 2.5 kHz (or higher). They considered that the above result was derived from the shape of the hypopharynx, which comprises the laryngeal tube and piriform fossa. The frequency regions we revealed are largely consistent with the results of these previous studies. In addition, considering the findings of these previous studies, the shape of the piriform fossa may be deeply involved in voice individuality in the 4–6 kHz frequency region.

From the results described in Section 4.2.2, we found a common individuality among the different vowels in the 1–2 kHz frequency region. However, the reason for this result remains unclear. In future, we plan to investigate the relationship between this frequency region and the vocal organs. We also found that each vowel exhibited unique individuality in frequency regions other than the 1–2 kHz and 4–6 kHz regions. Therefore, we expect that verification accuracy can be improved by utilizing these frequency regions observed in each vowel.

From the results presented in Section 4.3, we found that the 1–2 kHz and 4–6 kHz frequency regions included voice individuality that was robust to age-related changes. The evaluation of robustness was conducted using only vowel samples. In future, we will investigate voice individuality and its robustness for different manners of articulation such as fricatives, affricates, semivowels, stops, liquids, and nasals. In addition, we must consider more speaker voices obtained over a longer period to increase our understanding of voice individuality.

Voice biometrics technologies that are independent of age-related voice changes over a long period are expected to play an important role in various fields, e.g., speaker verification, identification of missing persons, and investigation of special fraud and kidnapping cases. In this sense, we believe that our findings represent a significant contribution to society and the voice biometrics field.

## 6. Conclusions

In this study, our objective was to verify whether there exists voice individuality that is unaffected by age-related changes by targeting adolescent Japanese speakers. We defined the immature and mature periods in vocal organ development as unstable and stable periods, respectively. In our initial experiment, we performed speaker verification tests across these two periods and evaluated voice features that are common to these periods using Fisher’s F-ratio. The experiment was performed using phrase and vowel samples. The speaker verification tests using the phrase samples demonstrated verification accuracy of 54% to 75%. For vowel samples, the verification accuracy ranged from 46% to 75%; however, most of them obtained verification accuracy of 60% or greater. These results suggest that it is possible to recognize speakers across the unstable and stable periods. In a subsequent evaluation of voice features using Fisher’s F-ratio, we observed voice features that are common to both the unstable and stable periods, and we found that both phrase and vowel samples contained voice individuality in the 4–6 kHz frequency region. For vowel samples, voice individuality was found in the 1–2 kHz frequency region regardless of the type of vowel. In addition, we found that each vowel exhibited unique frequency regions related to voice individuality. Furthermore, we found that the 1–2 kHz and 4–6 kHz frequency regions included voice individuality that was robust to age-related changes compared to the other frequency regions. These results suggest that there are invariant frequency features that are unaffected by age-related changes in the human voice.

In future, we plan to conduct additional experiments with more speakers over a longer period. In addition, we plan to construct a speaker verification model that focuses on the frequency regions revealed in this study. Furthermore, we will conduct similar investigations in languages other than Japanese, such as English and Chinese.

## Figures and Tables

**Table 1:** Details of voice actors.

Voice Actor	Gender	Unstable Period (Age)	Stable Period (Age)
Target M	Male	12, 13, 15, 16, 18	21, 22
Target F	Female	13, 14, 16, 17, 19	21, 22, 23
Non-target M1	Male	13, 14, 16, 17, 19	22, 23
Non-target M2	Male	12, 13, 15, 16, 18	22
Non-target F1	Female	14, 17, 19	21, 22, 23
Non-target F2	Female	17, 19	21

**Table 2:** Number of phrase and vowel samples.

	Unstable Period	Stable Period	Total
Phrase	47,041	22,709	69,750
Vowel /a/	4223	1535	5758
Vowel /i/	1277	591	1868
Vowel /u/	360	189	549
Vowel /e/	1266	613	1879
Vowel /o/	3254	1343	4597

## Data Availability

Not applicable.
